# Left Bundle Branch Area Pacing in Heart Failure: A Physiological Alternative to Conventional Biventricular Cardiac Resynchronization Therapy and a Step Towards Personalized Device Therapy

**DOI:** 10.3390/jpm16090463

**Published:** 2026-09-07

**Authors:** Ioanna Koniari, Andreas Gerakaris, Efthimia Kapsali, Stamatios Kanellopoulos, Georgia Xygka, Asimenia Katsea, Eftychia Petropoulou, Anastasia Mavromati, Rafail Koros, Georgios Leventopoulos, Angelos Perperis, Georgios Kollias, Periklis Davlouros

**Affiliations:** 1 Cardiology Department, University Hospital of Patras, 26504 Rion, Greece; agerakaris72@gmail.com (A.G.); efkapsali11@gmail.com (E.K.); s.kanellop@outlook.com (S.K.); georgia.xiga@gmail.com (G.X.); asimeniakatsea@icloud.com (A.K.); efipetro7@gmail.com (E.P.); natmavr@yahoo.gr (A.M.); korosraf@hotmail.com (R.K.); levent2669@gmail.com (G.L.); angelosperperis@msn.com (A.P.); pdav@upatras.gr (P.D.); 2 Liverpool Centre for Cardiovascular Science, Liverpool L14 3PE, UK; 3 Cardiology Department, Elisabethinen Hospital, 4020 Linz, Austria; dr.kollias@gmail.com

**Keywords:** heart failure, left bundle branch pacing, cardiac resynchronization therapy, personalized medicine, individualized patient selection, conduction system pacing

## Abstract

Heart failure remains a major cause of morbidity and mortality despite advances in guideline-directed medical therapy and device-based treatment. Cardiac resynchronization therapy delivered by conventional biventricular pacing constitutes an established therapeutic strategy for selected patients with heart failure with reduced ejection fraction and electrical desynchrony. Left bundle branch area pacing has emerged as a physiological pacing strategy that directly engages the conduction system, providing more effective electrical and mechanical resynchronization. Current evidence from observational studies, randomized pilot trials, and recent multicentre data suggest that left bundle branch pacing may achieve greater QRS narrowing, more favourable pacing thresholds, improved left ventricular ejection fraction, and reduced heart failure hospitalizations compared with conventional biventricular pacing in selected patients. This review summarizes the rationale, implantation criteria, patient selection, and current clinical evidence supporting left bundle branch pacing as an alternative pacing modality in heart failure patients. Despite encouraging available data, larger randomized trials with longer follow-up and broader patient populations are required to clarify long-term safety and to determine whether left bundle branch pacing should be adopted as a routine alternative to conventional cardiac resynchronization therapy. From a personalized medicine perspective, the choice between conduction system pacing and conventional biventricular pacing should be guided by individual patient characteristics, including conduction phenotype, QRS morphology and duration, myocardial scar burden, heart failure aetiology, coronary venous anatomy, and the chance of achieving confirmed conduction-system capture. This review therefore renders left bundle branch pacing within an individualized, phenotype-guided resynchronization strategy rather than as a universal replacement for biventricular pacing.

## 1. Introduction

Heart failure remains a leading cause of morbidity and mortality despite substantial advances in guideline-directed medical therapy and device-based pacing strategies. The syndrome is characterized by progressive impairment of left ventricular function, contributing to the initiation of compensatory neurohormonal, cellular, and structural responses. Although these mechanisms may initially preserve cardiac output, their chronic activation promotes adverse ventricular remodelling, such as changes in ventricular geometry, myocardial architecture, and electrical conduction. These maladaptive changes may contribute to delayed or heterogeneous ventricular activation, resulting in electrical and mechanical desynchrony, which further compromises left ventricular output and accelerates heart failure progression [1].

Cardiac resynchronization therapy (CRT), conventionally delivered by right ventricular pacing combined with left ventricular stimulation via a coronary sinus lead, constitutes a well-established treatment for selected patients with heart failure with reduced left ventricular ejection fraction (HFrEF) and electrical desynchrony. Large randomized trials have demonstrated that CRT reduces heart failure-related hospitalizations and enhances clinical outcomes in appropriately selected patients [2,3]. Nevertheless, a substantial proportion of HFrEF patients fail to demonstrate a significant clinical or echocardiographic response, rendering non-response an important limitation of conventional CRT [4]. Several factors may contribute to suboptimal response, such as myocardial scar burden, unfavourable coronary venous anatomy, non-optimal left ventricular lead position, high pacing thresholds, and phrenic nerve stimulation [5]. These limitations have increased interest in conduction system pacing as a more physiological alternative for achieving ventricular resynchronization [6].

Left bundle branch area pacing (LBBAP) has emerged as a novel physiological pacing strategy for ventricular resynchronization in heart failure patients. Pacing the left bundle branch immediately beyond the conduction block is described as an approach correlated with low and stable pacing outputs and direct engagement of the native conduction system, achieving QRS narrowing and more coordinated left ventricular activation [7]. LBBAP may provide a more physiological alternative to conventional biventricular pacing (BiV-P) in selected patients, in whom paced QRS morphology and the degree of resynchronization may be compromised by lead position, latency intervals, and interventricular timing optimization [8]. This review aims to provide a comprehensive overview of left bundle branch area pacing (LBBAP) as an alternative physiological pacing strategy in heart failure patients. Current clinical evidence, indications, procedural considerations, and criteria for successful implantation will be elucidated. By integrating current data and practical aspects of LBBAP, this review seeks to define its emerging role in contemporary heart failure management and cardiac resynchronization therapy.

Current recommendations continue to endorse conventional BiV-CRT as the first-line resynchronization strategy for patients who meet established guideline-based indications, given that its efficacy is supported by multiple large randomized trials. Conduction system pacing-based CRT, including LBBAP-CRT, is primarily recommended as a rescue strategy when effective coronary sinus lead implantation is unsuccessful and may be considered an alternative in carefully selected patients. Left bundle branch-optimized CRT, which combines LBBAP with coronary sinus left ventricular pacing, may be considered when either conduction system pacing or conventional BiV pacing yields a suboptimal electrocardiographic or clinical response. However, evidence supporting this hybrid approach remains largely observational, and its use should take into account procedural complexity, operator expertise, and individual patient risk (central illustration) [9,10].

Heart failure is, however, a heterogeneous syndrome, and the persistently high rate of non-response to conventional CRT indicates that a uniform device strategy is unlikely to serve all patients equally. Response is determined by patient-specific factors, such as the site and extent of conduction block, QRS morphology and duration, the burden and distribution of myocardial scar, heart failure aetiology, ventricular geometry, coronary venous anatomy, and rhythm status. Within this framework, LBBAP is best regarded not as a universal substitute for BiV-CRT, but as an additional therapeutic option that widens the range of resynchronization strategies available for the individual patient. A central aim of this review is therefore to synthesize the available evidence into a personalized framework that clarifies which patient phenotypes are most likely to benefit from LBBAP-CRT, which remain better served by conventional BiV-CRT, and in which patients a hybrid LOT-CRT approach may be appropriate.

### 1.1. Physiological Basis and Anatomical Rationale for Left Bundle Branch Area Pacing

#### 1.1.1. Anatomy of the His–Purkinje System and Normal Ventricular Activation

The His–Purkinje system is a specialized conduction network responsible for the rapid and synchronized activation of the ventricles. Following transmission through the atrioventricular node, the His bundle traverses the central fibrous body and extends along the membranous interventricular septum before dividing into the right and left bundle branches. The right bundle branch courses towards the right ventricular apex and free wall, whereas the left bundle branch forms a broad, fan-shaped subendocardial network that divides into anterior, posterior, and variably distinct septal fascicles. These fascicles connect with the distal Purkinje network, enabling the rapid distribution of electrical impulses throughout the left ventricular endocardium [6,11].

During normal ventricular activation, excitation emerges from multiple left ventricular endocardial breakthrough sites, particularly within the septal and apical regions, and rapidly propagates through the Purkinje network towards the lateral and basal myocardium. The near-synchronous activation of both ventricles generates a narrow QRS complex and coordinated mechanical contraction. This physiological activation pattern minimizes regional disparities in contraction timing, facilitates efficient ventricular pressure generation, and preserves the normal sequence of papillary muscle and ventricular wall activation [11].

#### 1.1.2. Pathophysiology of Left Bundle Branch Block

In left bundle branch block, conduction through the proximal left bundle branch or its distal fascicular network is interrupted or substantially delayed. Consequently, ventricular activation initially proceeds through the right bundle branch and is then transmitted slowly across the interventricular septum via transseptal myocardial conduction. This abnormal sequence results in atypical septal activation, with the posterolateral left ventricular wall generally activated last, thereby prolonging the QRS complex and increasing the dispersion of regional activation times. Human endocardial mapping has further demonstrated that the activation pattern in LBBB is heterogeneous and may vary according to the site of conduction block and the underlying myocardial substrate [12].

The abnormal electrical activation sequence produces both interventricular and intraventricular mechanical desynchrony. Premature septal contraction occurs while the lateral wall remains electrically inactive, whereas delayed contraction of the lateral wall may stretch the previously activated septum. These opposing regional forces can manifest as septal flash and apical rocking and are associated with impaired left ventricular pressure generation, increased regional wall stress, functional mitral regurgitation, reduced stroke work, and adverse ventricular remodelling. Ventricular desynchrony also diminishes myocardial mechanical efficiency; cardiac resynchronization therapy therefore seeks to restore a more coordinated contraction pattern and improve the relationship between myocardial work and oxygen consumption [13].

#### 1.1.3. Mechanistic Rationale for LBBAP Compared with Conventional BiV-CRT

Conventional biventricular cardiac resynchronization therapy seeks to compensate for delayed left ventricular activation by pacing the right ventricular endocardium and the left ventricular epicardium via a coronary sinus branch. Resynchronization is achieved through the interaction of two paced myocardial wavefronts rather than through complete restoration of the native His–Purkinje activation sequence. Although BiV-CRT improves morbidity, mortality, and ventricular remodelling in appropriately selected patients, its electrical effects are influenced by coronary venous anatomy, left ventricular lead position, myocardial scar, pacing latency, atrioventricular and interventricular timing, and the extent of fusion with intrinsic conduction. Moreover, epicardial left ventricular pacing reverses the physiological endocardial-to-epicardial activation sequence and may result in delayed activation of clinically important myocardial regions [1,4,5,9,13].

Left bundle branch area pacing is performed by advancing a pacing lead from the right ventricular aspect of the interventricular septum into the deep basal or mid-septal myocardium, in proximity to the left ventricular subendocardium. The term LBBAP encompasses direct left bundle branch pacing, left fascicular pacing, and left ventricular septal pacing. When the pacing stimulus captures the left bundle branch or one of its fascicles distal to the site of pathological conduction block, it can bypass the proximal conduction delay and propagate rapidly through the distal Purkinje network. This results in early, relatively homogeneous activation of the left ventricular endocardium, a short and stable stimulus-to-left ventricular activation time, and substantial narrowing of the paced QRS complex [6,7,14,15].

The principal physiological advantage of LBBAP lies in its direct or near-direct recruitment of the native left-sided conduction system, thereby reducing reliance on slow myocardial cell-to-cell conduction. Purkinje-mediated activation may enhance septal–lateral electrical coordination, decrease dispersion of ventricular activation, and promote more efficient mechanical contraction. Because the left ventricle is activated earlier through the left-sided conduction system and the right ventricle remains relatively delayed, the paced QRS complex commonly exhibits a terminal R or r′ wave in lead V1. Fusion with intrinsic right bundle branch conduction may further enhance biventricular synchrony. Compared with His-bundle pacing, the broader anatomical target of the left bundle branch area may also offer higher sensed R-wave amplitudes and lower, more stable capture thresholds [6,9].

**Figure 1:**
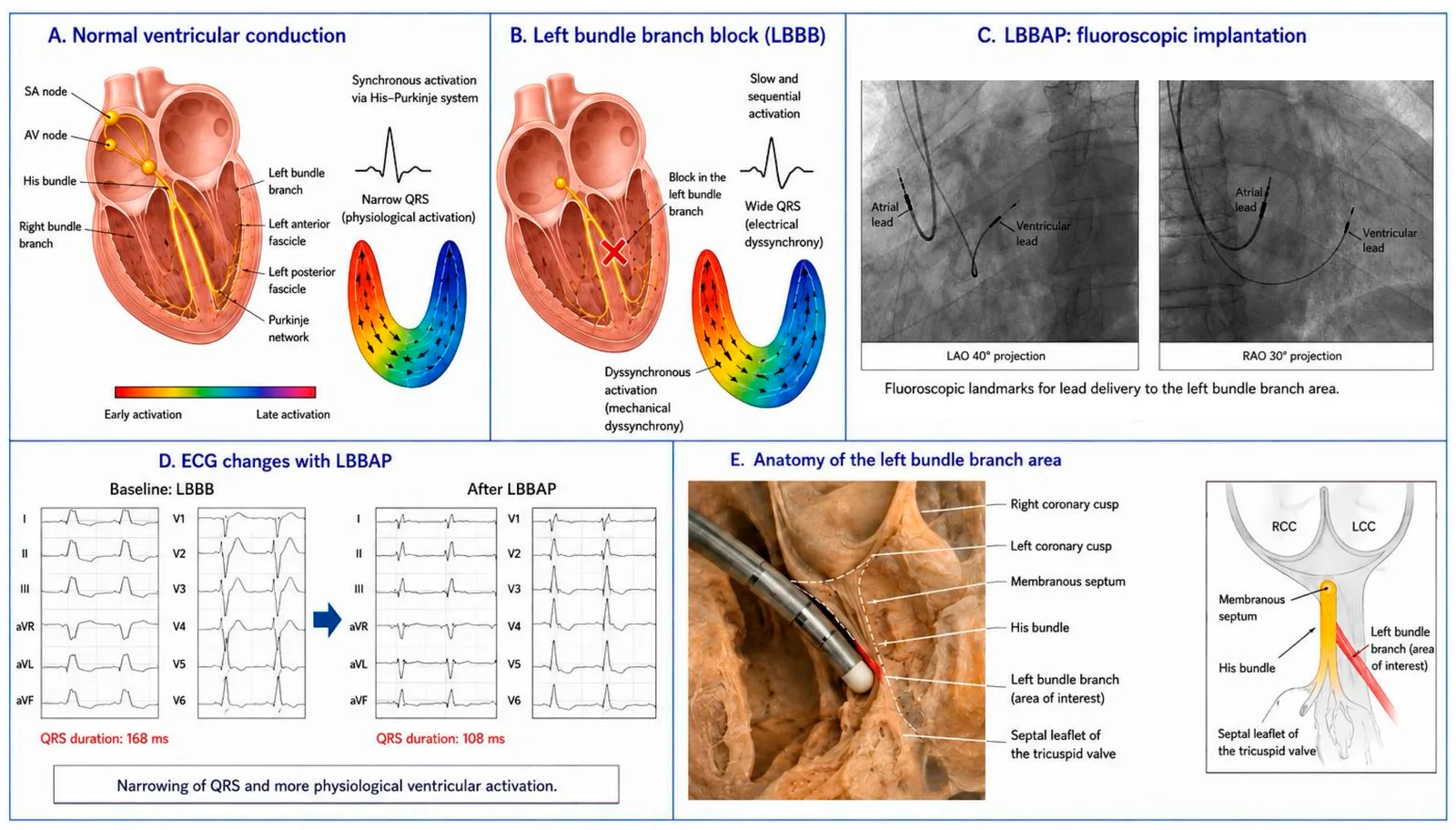
Normal conduction, LBBB pattern, and principle of left bundle branch area pacing (LBBAP). (**A**) Physiological activation via the His-Purkinje system with narrow QRS. (**B**) Conduction delay in LBBB with dyssynchronous ventricular activation and wide QRS. (**C**) Fluoroscopic landmarks for delivery of the pacing lead to the left bundle branch area (RAO 30° and LAO 40°). (**D**) Surface ECG before and after LBBAP showing QRS narrowing. (**E**) Relevant anatomy of the membranous septum and left bundle branch area targeted by the pacing lead.

Nevertheless, deep septal lead placement should not be regarded as inherently physiological. Its therapeutic efficacy depends on confirmed conduction-system capture, the anatomical location of the underlying conduction block, the extent of residual right ventricular activation delay, and the presence of conduction-system disease distal to the pacing site. Deep septal lead positioning should not be considered sufficient evidence of conduction-system capture. Accordingly, a multiparametric assessment that integrates paced QRS morphology, V6 R-wave peak time, stimulus-to-left ventricular activation time, output-dependent transitions, and conduction-system potentials is required to differentiate genuine left bundle branch capture from isolated left ventricular septal myocardial pacing [6,14,15,16,17,18]. Figure 1 summarizes the normal conduction-system anatomy, the pathophysiology of LBBB, and the mechanistic basis of resynchronization with LBBAP.

### 1.2. Criteria for Successful Left Bundle Branch Capture During LBBAP

Confirmation of left bundle branch (LBB) capture is an important step during left bundle branch area pacing (LBBAP), as deep septal lead positioning alone does not necessarily indicate direct engagement of the conduction system. Current assessment therefore relies on a combination of paced QRS morphology, ventricular activation timing, and changes in capture characteristics during pacing [6].

LBB capture typically results in rapid left ventricular activation followed by delayed right ventricular activation, producing a paced QRS with a terminal R/r′ wave in lead V1 [15]. Although this morphology supports appropriate lead positioning, it is not sufficient by itself to confirm conduction system capture. Additional evidence is provided by the stimulus-to-left ventricular activation time (Stim-LVAT), measured from the pacing stimulus to the R-wave peak in leads V5 or V6 [19]. A short and stable Stim-LVAT across different pacing outputs supports LBB capture [14,15]. An abrupt shortening of Stim-LVAT by ≥10 ms may provide further evidence of conduction system engagement [17], while demonstration of transitions between non-selective, selective, and myocardial capture during threshold testing provides additional confirmation [14,15].

Surface ECG criteria may further assist when clear capture transitions cannot be demonstrated. The V6 R-wave peak time (V6RWPT) is particularly useful: a cut-off of 74 ms is highly specific for LBB capture in patients with narrow QRS complexes or right bundle branch block, whereas a value ≤ 80 ms is highly specific in patients with LBBB, nonspecific intraventricular conduction delay, or wide-QRS escape rhythms [16]. Similarly, a V6–V1 interpeak interval > 44 ms strongly supports LBB capture, reflecting rapid left ventricular activation followed by delayed right ventricular activation [18].

Recording an LBB potential before the ventricular electrogram can provide additional supportive evidence, particularly in patients without baseline LBBB, although its absence does not exclude successful capture [14,15]. In selected cases, intracardiac findings such as retrograde His activation or anterograde distal LBB potentials may further strengthen the diagnosis [17,20]. Overall, no single parameter should be considered diagnostic in isolation; confirmation of LBB capture is most reliable when concordant electrocardiographic, activation-timing, and capture-transition criteria are present [6,17]. The main criteria used to support LBB capture are summarized in Table 1.

## 2. Patient Selection for Biventricular CRT and Left Bundle Branch Area Pacing in Heart Failure

Cardiac resynchronization therapy (CRT) has been shown to reduce heart failure hospitalizations and mortality in appropriately selected patients with HF, LBBB, and reduced left ventricular ejection fraction (LVEF). Conventional CRT is most frequently performed by biventricular pacing (BiVP), requiring the placement of a left ventricular lead through the coronary sinus into a suitable epicardial coronary venous branch, usually targeting the lateral or posterolateral left ventricular wall [6]. Although BiVP enhances ventricular synchrony and clinical outcomes in selected patients, it does not restore native His–Purkinje activation. Instead, resynchronization is achieved by fusion of paced wavefronts originating from the right ventricular endocardium and the left ventricular epicardium, with subsequent myocardial cell-to-cell propagation. This non-physiological activation pattern, along with the persistence of CRT non-response in a substantial proportion of patients, has increased interest in alternative pacing strategies that directly recruit the cardiac conduction system.

Conduction system pacing (CSP), particularly left bundle branch area pacing (LBBAP), has therefore emerged as a physiological approach to ventricular resynchronization. The 2025 ESC/EHRA clinical consensus statement identifies CSP as an increasingly adopted alternative to right ventricular pacing and in selected individuals to BiV-CRT, reflecting the expansion of evidence since the 2021 ESC pacing guidelines, when recommendations for LBBAP could not be established owing to limited data [9]. Depending on the extent of capture, LBBAP may be selective, with capture limited to the left bundle branch, or nonselective, with simultaneous capture of the left bundle branch and adjacent septal myocardial tissue. Resynchronization is ultimately achieved with selective LBBAP, rendering selective LBBAP an alternative CRT option in patients with heart failure.

Patient selection remains central when considering conventional BiV-CRT versus LBBAP. Established guideline indications continue to support BiV-CRT as the standard first-line resynchronization therapy in patients with symptomatic HF, LVEF ≤ 35%, sinus rhythm, LBBB morphology, and significant QRS prolongation despite guideline-directed medical therapy, particularly when QRS duration is ≥150 ms [4]. The expected benefit is less consistent in patients with non-LBBB morphology or less marked QRS duration, and therefore the strength of recommendation is lower in these patients [4]. The ESC/EHRA consensus similarly reports that the most robust evidence remains in favour of BiV-CRT for patients with classical CRT indications, especially those with LBBB and LVEF ≤ 35% [9].

At the same time, contemporary guidance indicates an expanding role for CSP-CRT in carefully selected patients. According to the ESC/EHRA consensus, CSP-CRT, particularly using LBBAP, may be used as an alternative to BiV-CRT in selected HF patients, but the absence of large randomized trials currently prevents its routine adoption over conventional BiV-CRT in daily practice [9]. Its role is especially relevant as a rescue therapeutic option when effective CRT cannot be achieved due to failure of coronary sinus lead implantation in a stable and suitable position. CSP-CRT may also be considered in patients who remain non-responders to BiV-CRT, particularly when persistent QRS prolongation suggests incomplete electrical resynchronization despite conventional biventricular pacing [4,9].

In patients with LVEF ≤ 35%, sinus rhythm, LBBB, QRS duration ≥ 150 ms, and NYHA class II–IV symptoms despite optimal medical therapy, LBBAP may therefore be considered when BiV-CRT cannot be effectively performed or when an individualized physiological pacing strategy is selected in an experienced CSP centre. For patients with LVEF ≤ 35% and non-LBBB morphology, especially when QRS duration is ≥150 ms, the role of LBBAP is less well defined and should be individualized, especially if conventional BiV-CRT is not feasible. In patients with non-LBBB morphology and QRS duration of 120–149 ms, the clinical benefit of LBBAP remains uncertain. Similarly, in patients with LVEF > 35% and no bradycardia pacing indication, evidence is still limited, although selected patients with LBBB and mildly reduced LVEF may represent a potential candidate group in whom CSP could contribute to preservation or improvement of ventricular function [9], as presented in Table 2.

Overall, BiV-CRT and LBBAP should currently be considered as complementary rather than competing therapeutic strategies. BiV-CRT remains the gold standard for patients with established CRT indications, whereas LBBAP constitutes a promising physiological alternative in selected cases, particularly after failed coronary sinus lead implantation, in CRT non-responders, or when direct correction of conduction delay is feasible. Careful patient selection, confirmation of conduction-system capture, and operator expertise remain significant for optimizing outcomes. Patient-specific structural evaluation should include ventricular function and morphology, septal anatomy, and the presence and distribution of myocardial scar. Echocardiography represents the primary imaging modality, while cardiac magnetic resonance (CMR) with late gadolinium enhancement can provide additional characterization of myocardial fibrosis and septal scar in selected patients. Cardiac CT and intraprocedural fluoroscopic or contrast-based assessment might also be useful when detailed anatomical definition is needed.

## 3. LBBAP as an Alternative to Conventional BiVP

Recent randomized evidence supports the idea that conduction system pacing can provide effective resynchronization compared with conventional BiV-CRT, although the available trials differ considerably in design and patient population as summarized in Table 3.

In the LBBAP-RESYNC trial, Wang et al. [22] carried out a small prospective randomized pilot study in a carefully selected group of patients with non-ischaemic cardiomyopathy, complete LBBB, sinus rhythm, NYHA class II–IV symptoms, and LVEF ≤ 40%. Patients with ischaemic cardiomyopathy, non-LBBB conduction patterns, and persistent atrial fibrillation were excluded, creating a relatively homogeneous cohort in which LBBB-related desynchrony was likely a major contributor to LV dysfunction. The study directly compared confirmed LBBAP-CRT with conventional BiV-CRT, and its main goal was to determine whether LBBAP-CRT led to greater improvement in LVEF at 6 months. In this setting, LBBAP-CRT produced significantly greater LVEF improvement than BiV-CRT, with a mean between-group difference of 5.6% (95% CI: 0.3–10.9; *p* = 0.039). It also showed larger reductions in LV end-systolic volume and NT-proBNP, while changes in NYHA class, 6 min walk distance, QRS duration, and CRT response were largely similar between groups. These results suggest that, in a selected non-ischaemic LBBB population, direct left bundle branch recruitment may produce more marked reverse remodelling than conventional epicardial LV pacing.

The CSP-SYNC trial by Žižek et al. [21] addressed a related but not identical question. Unlike LBBAP-RESYNC, CSP-SYNC was designed as a randomized non-inferiority study and enrolled patients with a Class I indication for CRT, defined by symptomatic heart failure despite optimal medical therapy, sinus rhythm, LVEF ≤ 35%, QRS duration ≥150 ms, and LBBB morphology. This population was broader and closer to guideline-defined CRT candidates, since it included both ischaemic and non-ischaemic cardiomyopathy. In addition, the intervention was left bundle branch area pacing rather than strictly confirmed LBBAP, reflecting a more pragmatic conduction system pacing approach. The primary endpoint was again LVEF change at 6 months, but the statistical goal was to show that LBBAP was not inferior to BiV pacing, rather than to prove superiority. LBBAP achieved this non-inferiority endpoint, with LVEF improving by 14% in the LBBAP group and 8.5% in the BiV group, and similar structural and functional improvements were seen during follow-up. Exploratory results, including higher EF normalization rates and a trend towards fewer heart failure hospitalizations, further supported the potential of LBBAP, though the trial was not powered to confirm superiority for clinical outcomes. Therefore, while Wang et al. provide evidence suggesting possible superiority of confirmed LBBAP in a narrowly selected non-ischaemic LBBB population, Žižek et al. support the non-inferiority of a broader LBBAP strategy in a more typical CRT population. Complementing these randomized data, Vijayaraman et al. [23] presented large-scale real-world evidence comparing LBBAP with conventional BiV pacing in CRT candidates. In this retrospective multicentre observational study, 1778 patients with LVEF ≤ 35% undergoing CRT for Class I or II indications were included across 15 international centres, of whom 797 received LBBAP and 981 received BiV pacing. LBBAP was associated with a significantly lower incidence of the composite endpoint of all-cause mortality or heart failure hospitalization compared with BiV pacing (20.8% vs. 28%; adjusted HR for BiV pacing vs. LBBAP: 1.495; 95% CI: 1.213–1.842; *p* < 0.001), mainly due to fewer heart failure hospitalizations rather than a statistically significant mortality difference. LBBAP also resulted in greater QRS narrowing, lower and more stable pacing thresholds, fewer procedural complications, and greater improvement in LVEF compared with BiV pacing. Echocardiographic response and hyper-response rates were higher with LBBAP as well, especially among patients with LBBB. These findings support the concept that more physiological ventricular activation may lead to improved structural and clinical outcomes compared with conventional BiV-CRT. However, because treatment allocation was not randomized and the pacing strategy depended on operator preference and institutional practice, these results remain subject to selection bias and residual confounding.

Earlier observational evidence from Wu et al. [24] further supports the physiological basis for LBBAP as a resynchronization approach. In this prospective, non-randomized, single-centre real-world study, consecutive patients with symptomatic heart failure, LVEF ≤ 40%, and typical LBBB defined strictly by Strauss criteria underwent CRT using His bundle pacing, LBBAP, or conventional BiV pacing. Although not randomized and therefore open to selection bias, the study provides useful mechanistic and clinical insight, since it directly compared conduction system pacing with BiV pacing in a population enriched with LBBB. Both His bundle pacing and LBBAP produced greater QRS narrowing than BiV pacing and were associated with larger improvements in LVEF at 1 year. In particular, LVEF increased by approximately 24% with LBBAP and 23.9% with His bundle pacing, compared with 16.7% with BiV pacing. The rates of LVEF normalization were also higher with LBBAP and His bundle pacing than with BiV pacing. Notably, LBBAP appeared to maintain many of the resynchronization advantages of His bundle pacing while providing more favourable pacing characteristics, such as lower and more stable capture thresholds and higher R-wave amplitudes. These results support the concept that direct recruitment of the His–Purkinje system may yield more effective electrical resynchronization than conventional BiV pacing, while suggesting that LBBAP may be more practical than His bundle pacing for long-term CRT delivery. Nevertheless, the non-randomized design, the small LBBAP sample and the predominance of non-ischaemic cardiomyopathy limit the generalizability of these findings, which should therefore be regarded as supportive mechanistic evidence rather than conclusive clinical proof.

Zhu et al. [25] further refine the interpretation of LBBAP by showing that outcomes can vary depending on the specific pacing phenotype achieved. In this prospective real-world cohort of 259 patients with reduced LVEF undergoing CRT, true LBBAP was associated with a notably lower risk of all-cause mortality or heart failure hospitalization than both BiV pacing and LVSP, mainly due to fewer heart failure hospitalizations. In contrast, LVSP did not offer the same advantage and was associated with higher mortality compared with BiV pacing. LBBAP also produced the highest echocardiographic response and super-response rates, suggesting that direct conduction system capture, rather than non-specific septal myocardial pacing, may be the principal determinant of favourable outcomes. This distinction is clinically important, since the potential benefit of physiological pacing may rely on precise confirmation of LBB capture. However, as this was a non-randomized cohort, these findings remain hypothesis-generating.

The CONSYST-CRT trial was a randomized non-inferiority study that compared conduction system pacing with BiVP in 134 patients with HFrEF and an indication for CRT. At 12 months, CSP was non-inferior to BiVP for the primary composite endpoint of all-cause mortality, cardiac transplantation, heart failure hospitalization, or an improvement in LVEF of less than 5 percentage points. However, crossover from CSP to BiVP occurred in 26.9% of patients, compared with 7.5% who crossed over from BiVP to CSP. This substantial crossover rate underscores important feasibility challenges, particularly among patients with intraventricular conduction disease, and supports the use of individualized or hybrid resynchronization strategies in selected patients [28].

A multicentre observational study by Li et al. showed that LBBAP is a feasible and effective strategy for CRT in patients with HFrEF and LBBB. LBBAP implantation was successfully performed in 81.1% of patients and resulted in significant electrical and mechanical resynchronization with QRS narrowing (*p* < 0.001) and improvement in both LVEF (*p* < 0.001) and NYHA functional class during a 6-month follow-up. Patients who received LBBAP achieved greater QRS narrowing on the ECG (58.0 vs. 12.5 ms; *p* < 0.001), a greater increase in LVEF (15.6% vs. 7.0%; *p* < 0.001) and higher rates of echocardiographic response (88.9% vs. 66.7%; *p* = 0.035) and super-response (44.4% vs. 16.7%; *p* = 0.007) compared with patients treated with conventional BiVP [27]. Similar results were observed in a study by Guo et al., in which LBBAP-CRT resulted in superior electrical resynchronization with greater narrowing of QRS duration (*p* < 0.0001) compared with conventional BiVP, along with a trend towards greater improvement in LVEF (*p* = 0.12), echocardiographic response (*p* = 0.43) and super-response rate (*p* = 0.09) [29].

A prospective, multicentre, observational study included 371 patients with HFrEF and compared the outcomes between LBBAP and BiVP as an initial implant strategy for CRT. Although LBBAP was associated with a significant reduction in HF hospitalizations (22.6% LBBAP vs. 39.5% BiVP; HR 0.607, 95% CI 0.397–0.927; *p* = 0.021), there was no significant difference in either all-cause mortality (5.5% LBBAP vs. 11.9% BiVP; *p* = 0.19) or in long-term complications (9.4% LBBAP vs. 15.2% BiVP; *p* = 0.146). However, in this study LBBAP was associated with shorter procedural duration (95 min LBBAP group vs. 129 min BiVP group; *p* < 0.001) and fluoroscopy time (12 min vs. 21.7 min; *p* < 0.001), shorter QRS duration (123.7 ± 18 msec LBBAP vs 149.3 ± 29.1 msec BiVP; *p* < 0.001) and higher post-procedural LVEF (34.1 ± 12.5% LBBAP vs. 31.4 ± 10.8% BiVP; *p* = 0.041) [26].

Although women have traditionally shown a better response to conventional CRT than men, a prospective multicentre registry by Tedrow et al. showed that men and women undergoing LBBAP had comparable clinical outcomes (*p* = 0.46). Although men treated with LBBAP had significantly fewer HF hospitalizations and lower all-cause mortality compared with those treated with conventional BiVP (*p* = 0.004), there was no statistically significant difference between women in the BiVP and LBBAP groups (*p* = 0.23). Based on these findings, LBBAP is an effective physiological CRT strategy that may potentially reduce sex differences in response and outcomes traditionally observed with BiVP [30].

The recently published multicentre, randomized LEFT-BUNDLE-CRT trial examined whether LBBAP-CRT was non-inferior to conventional BiV-CRT in 176 patients with guideline-based indications for CRT and typical LBBB. At six months, a favourable CRT response was observed in 94.6% of patients assigned to BiV-CRT and 89.7% of those assigned to LBBAP-CRT. Because the lower bound of the confidence interval fell below the prespecified non-inferiority margin of 0.90, non-inferiority could not be formally established. Nevertheless, both approaches yielded high response rates, with no significant differences in adverse events or heart failure hospitalizations between the groups [31].

### Interpretation of Inconsistent Trial Outcomes and Limitations of LBBAP-CRT

Despite its compelling electrophysiological rationale, the superiority of LBBAP over conventional BiV-CRT has not been consistently demonstrated in randomized trials. Importantly, failure to establish non-inferiority does not demonstrate that LBBAP is inferior; rather, it indicates that the study could not exclude a difference exceeding the prespecified non-inferiority margin with adequate statistical confidence. In the LEFT-BUNDLE-CRT trial, the observed response rates translated into non-response rates of 5.4% for BiV-CRT and 10.3% for LBBAP-CRT [31]. By comparison, the smaller CSP-SYNC trial reported echocardiographic response rates of 83% with LBBAP and 61% with BiV-CRT, corresponding to non-response rates of 17% and 39%, respectively; however, this difference did not reach statistical significance [21]. These apparently divergent estimates underscore that reported non-response rates are highly dependent on the study population, sample size, duration of follow-up, and criteria used to define CRT response.

Several methodological factors may explain why LEFT-BUNDLE-CRT failed to meet its prespecified non-inferiority endpoint. First, conventional BiV-CRT achieved an exceptionally high response rate of approximately 95%, creating a ceiling effect and leaving limited scope for LBBAP to demonstrate an additional benefit. Second, the trial enrolled only 176 patients and was designed to evaluate a six-month composite response rather than differences in mortality or heart failure hospitalization. The primary endpoint could be achieved through either an improvement in the clinical composite score or a reduction of at least 15% in left ventricular end-systolic volume; consequently, modest between-group differences in either component may have influenced the overall outcome. In addition, crossover occurred in 14.9% of randomized patients, potentially attenuating treatment-group differences in the intention-to-treat analysis. Finally, the LBBAP group had a slightly wider intrinsic QRS duration at baseline [31].

A physiological improvement in ventricular activation does not necessarily translate into superior clinical outcomes. QRS narrowing, reduced left ventricular activation time, and enhanced electrical synchrony represent mechanistic or surrogate endpoints rather than definitive measures of clinical benefit. Reverse remodelling and clinical outcomes are also influenced by myocardial scar burden and distribution, ischaemic aetiology, mitral regurgitation, right ventricular function, atrial fibrillation, medical therapy, and device programming. Furthermore, optimally delivered BiV-CRT is already highly effective in patients with typical LBBB. Accordingly, any incremental benefit of LBBAP over a well-positioned and appropriately programmed BiV system may be modest and detectable only in larger studies with longer follow-up.

The term LBBAP encompasses heterogeneous pacing phenotypes, including confirmed left bundle branch or fascicular capture and left ventricular septal myocardial pacing. Deep septal lead placement alone does not ensure direct conduction-system capture, and variability in implantation endpoints may result in differing degrees of resynchronization across studies. Moreover, LBBAP is most likely to correct a proximal and recruitable conduction block. Distal Purkinje disease, diffuse intraventricular conduction delay, septal scarring, or extensive myocardial pathology may preclude complete correction despite apparently satisfactory lead positioning. LBBAP may also result in delayed right ventricular activation, particularly in the absence of fusion with intrinsic right bundle conduction or when atrioventricular timing is suboptimal. The high rate of crossover from CSP to BiVP in CONSYST-CRT further demonstrates that both procedural feasibility and treatment effects are influenced by the underlying conduction substrate [28].

LBBAP avoids several limitations associated with coronary sinus leads, including dependence on venous anatomy, phrenic nerve stimulation, lead instability, and elevated left ventricular pacing thresholds. However, it introduces a distinct spectrum of risks related to the deep transseptal lead trajectory, including acute or delayed septal perforation, septal haematoma, injury to or fistula formation involving septal coronary vessels, new right bundle branch block or complete atrioventricular block, lead dislodgement, threshold elevation, loss of conduction-system capture, lead fracture, and uncertainty regarding the extraction of chronically implanted deep septal leads [6,9]. Among patients undergoing implantation for heart failure indications in the multicentre MELOS registry, the procedural success rate was 82.2%, while the overall complication rate was 11.7%; complications attributable specifically to the transseptal approach occurred in 8.3% of patients [32]. These observational findings primarily reflect early clinical experience and should not be construed as evidence from a direct randomized comparison with BiV-CRT. Conversely, conventional BiV-CRT remains subject to coronary sinus lead implantation failure, lead dislodgement, phrenic nerve stimulation, elevated pacing thresholds, and coronary venous injury [4,5]. Accordingly, LBBAP modifies rather than eliminates the procedural and long-term risks associated with CRT.

Collectively, the current evidence supports LBBAP-CRT as a promising complementary approach rather than an inherently superior alternative to BiV-CRT for all patients. Its effectiveness is likely to depend on careful patient selection, the anatomical location and recruitability of conduction block, confirmed conduction-system capture, operator expertise, and individualized device programming. Future multicentre randomized trials should apply standardized criteria for capture confirmation and programming, stratify participants according to LBBB phenotype, myocardial scar burden, and underlying aetiology, and be sufficiently powered to assess clinically meaningful long-term outcomes, including heart failure hospitalization, mortality, lead durability, and the feasibility of lead extraction.

## 4. Discussion

Available evidence indicates that left bundle branch area pacing is an important addition to the cardiac resynchronization therapy armamentarium, but it cannot yet be regarded as a universally superior alternative to conventional biventricular pacing. Although LBBAP may achieve marked QRS narrowing, low and stable capture thresholds, and favourable reverse remodelling, these electrical and echocardiographic findings are surrogate outcomes and do not consistently translate into reduced rates of heart failure hospitalization or mortality. Furthermore, randomized trials have generally been small, applied heterogeneous definitions of successful LBBAP and CRT response, and been conducted predominantly in experienced centres involving carefully selected patient populations.

Conversely, contemporary BiV-CRT performs very well in appropriately selected patients, setting a high benchmark for any newer strategy seeking to demonstrate comparable or superior outcomes [21,22,23,28,31].

The inconsistent findings across comparative trials should therefore be interpreted in light of both trial design and treatment implementation. In LEFT-BUNDLE-CRT, the high response rate observed with BiV-CRT may have produced a ceiling effect. Moreover, the modest sample size, crossover between treatment groups, reliance on a composite six-month response endpoint, and variability in achieving confirmed conduction-system capture reduced the study’s ability to demonstrate non-inferiority [31]. LOT-CRT and BiV pacing produced greater improvements in left ventricular dP/dtmax than LBBAP alone, although LOT-CRT achieved the greatest QRS shortening [33].

A key conceptual limitation is that the presence of a deeply positioned septal lead does not, in itself, confirm capture of the left bundle branch. The term LBBAP encompasses several pacing modalities, including true left bundle branch pacing, fascicular pacing, and left ventricular septal myocardial pacing. These modalities may generate distinct ventricular activation patterns and may not provide equivalent degrees of resynchronization. Confirmation of conduction-system capture therefore requires a multiparametric evaluation that considers output-dependent transitions, stimulus-to-left ventricular activation time, V6 R-wave peak time, paced QRS morphology, and the presence of conduction-system potentials [6,14,15,16,17,18]. Accordingly, variability in implantation techniques and criteria for confirming conduction-system capture may attenuate the observed treatment effect and hinder valid comparisons across studies.

### 4.1. Practical Limitations, Non-Response, and Patient Selection for LBBAP-CRT

The translation of LBBAP-CRT into routine clinical practice remains constrained by procedural complexity, anatomical variability, and operator dependence. The learning curve encompasses selection of an appropriate septal entry site, maintenance of a suitable lead trajectory, controlled advancement through the interventricular septum, confirmation of conduction-system capture, and avoidance of excessively deep lead deployment. In the multicentre MELOS registry, implantation success among patients treated for heart failure indications was 82.2%, demonstrating that successful implantation cannot be assumed even in experienced centres [32]. Marked ventricular dilatation, septal hypertrophy or fibrosis, an unfavourable lead trajectory, distal conduction-system disease, and difficulty achieving stable capture may all contribute to procedural failure. Moreover, the lack of standardized implantation workflows, reporting criteria, and device-programming protocols limits reproducibility beyond high-volume centres [6,9,32].

LBBAP introduces a distinct risk profile rather than eliminating the complications associated with CRT. Reported transseptal lead-related complications include acute or delayed septal perforation, septal haematoma, coronary artery injury or spasm, coronary fistula formation, transient or permanent right bundle branch injury, atrioventricular block, lead dislodgement, threshold elevation, loss of conduction-system capture, helix entrapment, and lead damage or fracture [32]. Although conventional BiV-CRT is associated with coronary sinus cannulation failure, venous dissection or perforation, phrenic nerve stimulation, elevated pacing thresholds, and left ventricular lead instability, its long-term lead performance and extraction procedures are substantially better established. In contrast, uncertainties persist regarding the chronic integrity of LBBAP leads within the contracting interventricular septum, late changes in capture thresholds, progression of conduction-system disease, and the feasibility and safety of extracting deeply implanted septal leads after prolonged use. Recent observational studies have reported stable electrical parameters and relatively low rates of lead-related complications during intermediate-term follow-up. Nevertheless, these cohorts included patients with heterogeneous pacing indications and do not adequately address very long-term lead integrity, the technical challenges of extracting chronically implanted deep-septal leads, or outcomes specifically in patients receiving CRT [34,35].

Non-response remains clinically relevant with both strategies. In LEFT-BUNDLE-CRT, the six-month non-response rate was 10.3% with LBBAP-CRT and 5.4% with BiV-CRT, and non-inferiority was not formally demonstrated [31]. Potential mechanisms underlying non-response to LBBAP include failure to achieve or sustain genuine conduction-system capture, persistent distal conduction delay, residual right ventricular activation delay, septal or lateral-wall myocardial scar, advanced ventricular remodelling, non-LBBB conduction patterns, inadequate effective pacing, atrial fibrillation, and suboptimal atrioventricular programming. Accordingly, LBBAP should not be considered capable of overcoming all mechanisms of CRT non-response. Although it modifies the pathway of ventricular activation, it cannot reverse advanced myocardial disease or compensate for inappropriate patient selection.

Accordingly, the best-supported candidates for LBBAP-CRT are patients with symptomatic HFrEF, sinus rhythm, typical LBBB with a QRS duration of at least 150 ms, and a high likelihood of achieving confirmed conduction-system capture, particularly when coronary sinus lead implantation is unsuccessful or fails to provide adequate electrical correction [9,10,21,22,23]. Evidence remains limited in patients with atrial fibrillation, ischaemic cardiomyopathy, extensive septal scarring, non-LBBB patterns or nonspecific intraventricular conduction delay, and severe ventricular dilatation. These populations have been underrepresented in randomized trials; consequently, findings from highly selected cohorts with LBBB should not be extrapolated uncritically to routine clinical practice.

### 4.2. Implications for Personalized Medicine

These considerations place LBBAP-CRT firmly within the framework of personalized medicine. Rather than replacing conventional BiV-CRT, LBBAP allows the resynchronization strategy to be matched to the individual electrical and myocardial substrate. Patients with typical LBBB, a QRS duration of at least 150 ms, sinus rhythm, and limited septal scarring are the most likely to benefit from direct recruitment of the conduction system, whereas patients with non-LBBB conduction patterns, nonspecific intraventricular conduction delay, extensive septal fibrosis, or distal conduction-system disease can be better served by conventional BiV-CRT or by a hybrid LOT-CRT approach. Individualization should extend beyond preprocedural selection to the procedure itself: multiparametric confirmation of conduction-system capture, incorporating output-dependent transitions, stimulus-to-left ventricular activation time, V6 R-wave peak time, and paced QRS morphology, converts an anatomically defined lead position into a physiologically verified, patient-specific therapeutic endpoint. Individualized programming of atrioventricular and interventricular timing, together with structured follow-up of capture stability, further can tailor therapy to the underlying substrate. Prospective stratification according to conduction phenotype, scar burden, aetiology, and rhythm status, ideally supported by cardiac magnetic resonance imaging, electrocardiographic imaging, and echocardiographic assessment of mechanical synchrony, constitutes the most productive route towards patient-level prediction of response and towards a personalized approach to cardiac resynchronization.

Future research should focus on adequately powered, multicentre randomized trials comparing LBBAP-CRT with optimized BiV-CRT based on standardized definitions of implantation success, selective and non-selective left bundle branch capture, left ventricular septal pacing, crossover, and clinical responsiveness. Such studies should include independent electrocardiographic and imaging judjment, stratification according to conduction phenotype, myocardial scar burden, and heart failure aetiology, as well as long-term assessment of mortality, heart failure hospitalization, quality of life, lead durability and cost-effectiveness. The potential role of LOT-CRT should also be prospectively evaluated in patients with incomplete electrical or mechanical correction following either LBBAP or BiV-CRT. Until these data become available, LBBAP-CRT and LOT-CRT should be regarded as complementary, expertise-dependent approaches requiring individualized patient selection, rather than routine alternatives for BiV-CRT [9,10]. Early international experience with LOT-CRT established its procedural feasibility, but the available evidence was derived from non-randomized studies. Similarly, CSPOT assessed acute haemodynamic effects rather than long-term clinical outcomes. Consequently, the optimal indications for adding a coronary venous lead and the comparative effects of LOT-CRT on hospitalization and mortality remain uncertain [33,36]. The mechanisms, advantages, limitations, patient selection, procedural risks, and unresolved questions for BiV-CRT and LBBAP-CRT are compared in Table 4.

## 5. Conclusions

Overall, the available evidence supports LBBAP as a feasible and promising complementary approach to cardiac resynchronization, particularly in carefully selected patients with typical LBBB and in those for whom conventional BiV-CRT is unsuccessful or yields an inadequate response. Nevertheless, BiV-CRT remains the established standard of care for patients meeting current guideline-based indications, and LBBAP should not yet be considered a routine superior alternative. Its clinical efficacy is influenced by the underlying myocardial substrate, the achievement and long-term maintenance of conduction-system capture, operator expertise, and individualized device programming. Larger, multicentre randomized trials with extended follow-up and sufficient power to assess clinical outcomes are needed to compare the effects of these strategies on heart failure hospitalization, mortality, procedural complications, lead durability, extractability, and to clarify the roles of LBBAP-CRT and LOT-CRT in broader patient populations. Framed in terms of personalized medicine, the principal message of this review is that the optimal resynchronization strategy is patient-specific: LBBAP-CRT, BiV-CRT, and LOT-CRT should be regarded as complementary options, to be selected according to conduction phenotype, myocardial substrate, anatomical constraints, and the likelihood of achieving and maintaining conduction-system capture, rather than as competing universal standards.

## Figures and Tables

**Table 1:** Criteria of effective LBBP capture.

Criteria	Description	1st Author (Year)
1. Paced QRS morphology: The paced ECG should show a Right Bundle Branch Block (RBBB) pattern—meaning the left ventricle is activated first.	When the lead is placed transeptally from the right ventricular (RV) septum to LV septal subendocardium in the LBB region, the paced QRS morphology changes from an LBBB to a right bundle branch block (RBBB) pattern, as the LV is activated earlier than the RV. In addition to the RBBB pattern, the capture of the proximal left anterior or posterior fascicle may display in the ECG as left posterior or anterior fascicular block morphology.	Huang, W., et al. (2017) [7]
		Chen, X., et al. (2019) [15]
2. Identification of a Left Bundle Branch (LBB) potential: Recording an LBB potential (a sharp signal between atrial and ventricular signals) at the lead site, especially if intrinsic rhythm is present.	In patients with non-LBBB during intrinsic rhythm, LBB potentials should always be recorded from the LBBP lead, with the potential-to-ventricle interval of 20–30 ms, to help confirm lead position and the level of conduction block.	Chen, X., et al. (2019) [15]
	However, in patients with LBBB, LBB potentials can only be recorded during restoration of left bundle conduction by means of HBP or under other conditions.	Chen, X., et al. (2019) [15]
3. Short and consistent Stimulus-to-Left Ventricular Activation Time (Stim-LVAT): The time from the pacing spike to the peak of the R wave in leads V4–V6 should be short and constant at both low and high pacing outputs.	Pacing stimulus-to-left ventricular activation time (Stim-LVAT) is defined as the interval from the pacing stimulus to the peak of the R wave and is often used to reflect the lateral precordial myocardium depolarization time in leads V4–V6. Stim-LVAT that shortens abruptly with increasing output or remains shortest. and constant both at low and high outputs suggests LBB capture, which is also shorter than that in HBP.	Chen, X., et al. (2019) [15]
		Jastrzębski, M., et al. (2021) [16]
4. Demonstration of selective or nonselective LBB capture: - Selective capture: Only the conduction system is captured, shown by a discrete local electrogram. - Nonselective capture: Both conduction system and myocardium are captured together.	Selective LBBP, capturing only the LBB as a direct LBB capture sign, can be demonstrated with a discrete local component separate from the stimulus artifact on the unipolar electrogram (EGM) from the LBBP lead. Capturing both the LBB and the adjacent local septal myocardium results in nonselective LBBP with no discrete local EGM and electrocardiogram morphology slightly different from that of selective LBBP.	Chen, X., et al. (2019) [15]
		Wu, S., et al. (2019) [20]
5. Direct evidence of LBB capture: If recorded, retrograde His potentials or distal LBB potentials appearing with a short stimulus-to-potential interval confirm direct capture—although this is not mandatory in routine practice.	The interval between the pacing stimulus and the retrograde His potential or anterograde distal LBB potential recorded with an additional lead/catheter during LBBP can be used to confirm LBB capture, but it need not be routinely used in clinical practice.	Wu, S., et al. (2021) [17]
6. V6-V1 interpeak interval > 44 ms.		Jastrzebski, M., et al. (2022) [18]

**Table 2:** Recommendation/Consensus.

Clinical Scenario	HRS/APHRS/LAHRS 2023 Recommendation [4]	ESC/EHRA 2025 Consensus [16]
LVEF ≤ 35%, sinus rhythm, LBBB, QRS ≥ 150 ms, NYHA II–IV	BiV-CRT has a Class I indication. CSP/LBBAP is reasonable if effective BiV-CRT cannot be achieved, and may be considered as an alternative in selected cases.	BiV-CRT remains the reference standard with the strongest evidence. CSP-CRT/LBBAP may be appropriate in selected patients, but routine replacement of BiV-CRT is not yet supported by large randomized trials.
Failed coronary sinus LV lead implantation	CSP/LBBAP is reasonable when effective CRT cannot be achieved because of anatomical or functional limitations.	CSP-CRT is supported as a rescue strategy when a coronary sinus lead cannot be placed in a stable or effective position.
BiV-CRT non-responder with persistent wide QRS	CSP/LBBAP may be considered as an alternative or upgrade strategy, although supporting evidence is less robust than for conventional CRT.	CSP-CRT may be appropriate in selected BiV-CRT non-responders, particularly when persistent QRS prolongation suggests incomplete electrical resynchronization.
LVEF ≤ 35%, non-LBBB, QRS ≥ 150 ms, NYHA II–IV	CPP/CSP has a Class IIb recommendation in selected non-LBBB patients.	Evidence for CSP-CRT in non-LBBB morphology is less consistent; no clear superiority over BiV-CRT has been established.
Non-LBBB, QRS 120–149 ms	Limited Class IIb support in selected symptomatic patients; overall evidence remains limited.	Considered an area of uncertainty because data are insufficient to establish clear CSP benefit.
LVEF 36–50%, LBBB, QRS ≥ 150 ms, NYHA II–IV	CPP, including CRT or CSP, may be considered to maintain or improve LVEF.	Selected patients with mildly reduced LVEF and LBBB may benefit, but larger randomized studies are needed.

**Table 3:** LBBAP compared to conventional BiV-CRT.

Study	Population (n)	Follow-Up (n)	Post LVEF LBBP vs. BiVP (%)	Mortality (n)	QRS Width LBBP vs. BiVP (ms or %)	HF Hospitalization (%)	Key Findings
Zizek et al. [21]	62	12	ΔLVEF 14.0%; 95% CI: 11.2–16.8 vs. 8.5%; 95% CI: 5.6–11.2	N/A	ΔQRS −33 ms; 95% CI: −40 to −26 vs. −32 ms; 95% CI: −39 to −25	6.5% vs. 22.6% HR 0.26; 95% CI: 0.05–1.26; *p* = 0.09	LBBAP was non-inferior to BiV pacing in improving LVEF and provided similar structural and electrical remodelling
Wang et al. [22]	40	6	ΔLVEF LBBP 21.08 ± 1.91 ΔLVEF BiVP 15.62 ± 1.94 *p* = 0.039	No difference	LBBP 174.6 ± 14.3 ms to 131.5 ± 12.5 ms BiVP 174.7 ± 14.1 ms to 136.6 ± 12.9 ms)	No difference	LBBP-CRT demonstrated greater LVEF improvement than BiVP-CRT in heart failure patients with nonischemic cardiomyopathy and LBBB
Vijayaraman et al. [23]	1778	6	27 ± 6% to 41 ±13% (*p* < 0.001) with LBBAP compared with an increase from 27 ± 7% to 37 ±12% (*p* < 0.001) with BVP	21% LBBP 28% BiVP HR 1.495; 95% CI 1.213–1.842; *p* < 0.001	LBBP 128 ± 19 ms vs. BiVP 144 ± 23 ms *p* < 0.001	21% LBBP vs. 28% BiVP HR 1.495; 95% CI 1.213–1.842 *p* < 0.001	LBBAP improved clinical outcomes compared with BVP in patients with CRT indications and may be a reasonable alternative to BVP.
Wu et al. [24]	137	12	ΔLVEF LBBP 24% vs. BIVP 16.7%, *p* = 0.015	N/A	LBBP ΔQRS 56 ms BiVP ΔQRS 26 ms	N/A	LBBP improvements were significantly greater than those seen in patients treated with BVP
Zhu et al. [25]	259	24	33.7 ± 7.4 LBBP 28.9 ± 6.1 BIV	LBBP 7.4% vs. BiVP 41.2%; adjusted hazard ratio [aHR], 0.22 [0.08–0.57]; *p* = 0.002	LBBP 159.5 ± 36.5 ms BiVP 169.8 ± 23.6 ms	LBBP 4.4% vs. BiVP 36.6% *p* < 0.001	LBBP yields long-term clinical outcomes superior to those of BiVP
Diaz et al. [26]	371	12	34.1 ± 12.5% vs. 31.4 ± 10.8%; *p* = 0.041	LBBP 5.5% vs. BiVP 11.9% *p* = 0.19	LBBP 123.7 ± 18 ms vs. BiVP 149.3 ± 29.1 ms *p* < 0.001	LBBP 22.6% vs. BiVP 39.5% HR: 0.607 95% CI: 0.397–0.927 *p* = 0.021	LBBAP as an initial CRT strategy resulted in a lower risk of HF-related hospitalizations compared to BiVp.
Li et al. [27]	37	6	28.8 ± 4.5% to 44.3 ± 8.7%, *p* < 0.001	N/A	LBBP 121.8 ± 10.8 ms vs. BiVP 178.2 ± 18.8 *p* < 0.001	N/A	promising alternative resynchronization approach to BVP.
Pujol et al. [28]	134	12	ΔLVEF 15.3 ± 10.2 13.8 ± 10.1 *p* = 0.28	LBBP 18.5% vs. BiVP 31.6% *p* = 0.001 Combined endpoint	ΔQRS LBBP −50 ± 23 BiVP −45 ± 23 *p* < 0.001	LBBP 18.5% vs. BiVP 31.6% *p* = 0.001 Combined endpoint	CSP was noninferior to BiVP
Guo et al. [29]	21	6	LBBP-CRT group (50.9 ± 10.7% vs. 44.4 ± 13.3%, *p* = 0.12)	No difference	LBBP 167.7 ± 14.9 ms to 111.7 ± 12.3 ms (*p* < 0.0001) BiVP 163.6 ± 13.8 ms to 130.1 ± 14.0 ms (*p* < 0.0001)	No difference	LBBP-CRT can dramatically improve the electrical synchrony
Tedrow et al. [30]	539	13.2	N/A	Included in composite; not reported separately	N/A	Composite HFH/death: men 29.9% LBBAP vs. 46.5% BiVP; *p* = 0.004; women 24.1% vs. 36.2%; *p* = 0.23	LBBAP outcomes were similar in men and women; composite events were lower vs. BiVP in men, but not significantly different in women.
Cano et al. (LEFT-BUNDLE-CRT) [31]	176	12	N/A	No significant difference	N/A	No significant difference	LBBAP-CRT did not meet non-inferiority vs. BiVP-CRT. Six-month CRT response: 89.7% vs. 94.6% (RR 0.95; 95% CI 0.88–1.02).

N/A: Non Aplicable.

**Table 4:** Comparison of biventricular cardiac resynchronization therapy and left bundle branch area pacing for cardiac resynchronization.

Clinical Domain	Conventional BiV-CRT	LBBAP-CRT
Physiological mechanism	The conventional biventricular cardiac resynchronization therapy method works by combining the right ventricular endocardial and coronary sinus left ventricular epicardial wavefronts. The cardiac resynchronization therapy depends partly on cell-to-cell conduction and does not fully reproduce native His–Purkinje activation.	The left bundle branch area pacing method uses a septal lead to capture the left bundle branch or its fascicles distal to the site of block. This enables Purkinje-mediated left ventricular activation. The physiological benefit of left bundle branch area pacing depends on confirmed conduction-system capture rather than septal myocardial capture alone.
Current evidence and recommendations	Biventricular cardiac resynchronization therapy is established as a first-line cardiac resynchronization therapy for guideline-supported indications. Large randomized trials have demonstrated reductions in heart-failure hospitalization and mortality.	Left bundle branch area pacing is a rescue strategy when effective conventional biventricular cardiac resynchronization therapy cannot be achieved. It is also an alternative in selected cardiac resynchronization therapy candidates. However, evidence is expanding. Routine replacement of conventional biventricular cardiac resynchronization therapy is not supported because randomized data remain limited and inconsistent.
Principal advantages	Biventricular cardiac resynchronization therapy has a mature evidence base, standardized implantation and programming, extensive operator experience and established long-term lead performance and extraction pathways.	Left bundle branch area pacing results in greater QRS narrowing, direct or near-direct recruitment of the conduction system, physiological endocardial activation, low and generally stable capture thresholds and avoidance of coronary venous anatomical limitations.
Principal limitations	Biventricular cardiac resynchronization therapy is dependent on suitable coronary venous anatomy and an adequate epicardial target. It also has non-physiological epicardial-to-endocardial activation, possible phrenic stimulation, high thresholds, lead instability or scar at the target site.	Left bundle branch area pacing has limitations such as septal lead placement, which does not invariably produce true left bundle branch capture. Implantation, capture confirmation and programming are operator-dependent. Procedural definitions remain incompletely standardized and long-term extraction experience is limited.
Potential mechanisms of non-response	Non-response to conventional biventricular cardiac resynchronization therapy can occur due to suboptimal coronary sinus lead location, lateral or posterolateral scar inadequate electrical separation, low effective biventricular pacing percentage, atrial fibrillation suboptimal AV/VV programming and advanced myocardial disease.	Non-response to left bundle branch area pacing can occur due to failure to achieve or maintain left bundle branch capture, left ventricular septal pacing, distal Purkinje disease, septal or lateral-wall scar, residual right ventricular delay, incomplete correction of interventricular conduction delay, atrial fibrillation, suboptimal AV programming and advanced remodelling.
Procedure-specific risks	Procedural risks associated with conventional biventricular cardiac resynchronization therapy include coronary sinus cannulation failure, dissection or perforation, left ventricular lead dislodgement, phrenic nerve stimulation, high thresholds and complications related to an additional transvenous lead.	Left bundle branch area pacing has risks such as delayed septal perforation, septal haematoma, coronary injury or fistula, right bundle branch injury or AV block, lead dislodgement threshold elevation, loss of capture, helix entrapment, lead damage and uncertain chronic extraction risk.
Established candidates	The best candidates for conventional biventricular cardiac resynchronization therapy are symptomatic heart failure with reduced ejection fraction patients despite guideline-directed therapy, left ventricular ejection fraction ≤ 35%, sinus rhythm, typical left bundle branch block and QRS duration ≥ 150 ms.	The best candidates for bundle branch area pacing are symptomatic heart failure with reduced ejection fraction with sinus rhythm, broad typical left bundle branch block, QRS duration ≥ 150 ms, limited septal scar, preserved distal conduction and a high probability of confirmed conduction-system recruitment, particularly after failed or suboptimal coronary sinus lead implantation.
Patients with less predictable benefit	Patients with non-left bundle branch block morphology, QRS duration < 150 ms, extensive myocardial scar, severe right ventricular dysfunction or inability to maintain a high effective pacing percentage may have less predictable benefits from conventional biventricular cardiac resynchronization therapy.	Patients with non-left bundle branch block or nonspecific interventricular conduction delay, extensive ischaemic or septal scar, distal conduction disease, severe ventricular dilatation, persistent atrial fibrillation without reliable pacing, and heart failure with preserved ejection fraction may have less predictable benefits from left bundle branch area pacing.
Role of LOT-CRT	Biventricular cardiac resynchronization therapy remains appropriate when satisfactory electrical and mechanical resynchronization is achieved.	Left bundle branch area pacing combined with venous left ventricular pacing, also known as LOT-CRT, may be considered when left bundle branch area pacing alone leaves residual delay or when conventional biventricular cardiac resynchronization therapy provides incomplete correction. However, evidence is currently observational or based on haemodynamic studies.
Major unresolved questions	There are still unanswered questions regarding the optimal management of non-left bundle branch block patients, borderline QRS duration and persistent cardiac resynchronization therapy non-response.	Other unresolved questions include confirmation of left bundle branch capture performance outside high-volume centres, optimal lead design and programming, long-term integrity and extraction cost-effectiveness and effects on mortality and hospitalization, in adequately powered multicentre trials.

## Data Availability

The original contributions presented in this study are included in the article. Further inquiries can be directed to the corresponding authors.

## Abbreviations

AF	Atrial fibrillation
AV	Atrioventricular
BiV	Biventricular
BiV-CRT	Biventricular cardiac resynchronization therapy
BiVP	Biventricular pacing
CI	Confidence interval
CRT	Cardiac resynchronization therapy
CS	Coronary sinus
CSP	Conduction system pacing
CSP-CRT	Conduction system pacing for cardiac resynchronization therapy
CSPOT	Conduction System Pacing Optimized Therapy
ECG	Electrocardiogram
EF	Ejection fraction
EHRA	European Heart Rhythm Association
ESC	European Society of Cardiology
HF	Heart failure
HFrEF	Heart failure with reduced ejection fraction
HBP	His bundle pacing
HR	Hazard ratio
IVCD	Intraventricular conduction delay
LAO	Left anterior oblique
LBB	Left bundle branch
LBBAP	Left bundle branch area pacing
LBBAP-CRT	Left bundle branch area pacing for cardiac resynchronization therapy
LBBB	Left bundle branch block
LBBP	Left bundle branch pacing
LOT-CRT	Left bundle branch-optimized cardiac resynchronization therapy
LV	Left ventricle/left ventricular
LV dP/dtmax	Maximal rate of rise in left ventricular pressure
LVAT	Left ventricular activation time
LVEF	Left ventricular ejection fraction
LVSP	Left ventricular septal pacing
MELOS	Multicentre European Left Bundle Branch Area Pacing Outcomes Study
NT-proBNP	N-terminal pro-B-type natriuretic peptide
NYHA	New York Heart Association
QRS	QRS complex
RAO	Right anterior oblique
RBBB	Right bundle branch block
RV	Right ventricle/right ventricular
SA	Sinoatrial
Stim-LVAT	Stimulus-to-left ventricular activation time
V6RWPT	V6 R-wave peak time

## References

[1] Thomas G., Kim J., Lerman B.B. (2019). Improving Cardiac Resynchronisation Therapy. Arrhythmia Electrophysiol. Rev..

[2] Anand I.S., Carson P., Galle E., Song R., Boehmer J., Ghali J.K., Jaski B., Lindenfeld J., O’Connor C., Steinberg J.S. (2009). Cardiac resynchronization therapy reduces the risk of hospitalizations in patients with advanced heart failure: Results from the Comparison of Medical Therapy, Pacing and Defibrillation in Heart Failure (COMPANION) trial. Circulation.

[3] Moss A.J., Hall W.J., Cannom D.S., Klein H., Brown M.W., Daubert J.P., Estes N.A.M., Foster E., Greenberg H., Higgins S.L. (2009). Cardiac-resynchronization therapy for the prevention of heart-failure events. N. Engl. J. Med..

[4] Gerra L., Bonini N., Mei D.A., Imberti J.F., Vitolo M., Bucci T., Boriani G., Lip G.Y. (2025). Cardiac resynchronization therapy (CRT) nonresponders in the contemporary era: A state-of-the-art review. Heart Rhythm.

[5] Jastrzebski M., Wilinski J., Fijorek K., Sondej T., Czarnecka D. (2013). Mortality and morbidity in cardiac resynchronization patients: Impact of lead position, paced left ventricular QRS morphology and other characteristics on long-term outcome. Europace.

[6] Burri H., Jastrzebski M., Cano Ó., Čurila K., de Pooter J., Huang W., Israel C., Joza J., Romero J., Vernooy K. (2023). EHRA clinical consensus statement on conduction system pacing implantation: Executive summary. Endorsed by the Asia-Pacific Heart Rhythm Society (APHRS), Canadian Heart Rhythm Society (CHRS) and Latin-American Heart Rhythm Society (LAHRS). Europace.

[7] Huang W., Su L., Wu S., Xu L., Xiao F., Zhou X., Ellenbogen K.A. (2017). A Novel Pacing Strategy with Low and Stable Output: Pacing the Left Bundle Branch Immediately Beyond the Conduction Block. Can. J. Cardiol..

[8] Herweg B., Ali R., Ilercil A., Madramootoo C., Cutro R., Weston M.W., Barold S.S. (2010). Site-specific differences in latency intervals during biventricular pacing: Impact on paced QRS morphology and echo-optimized V-V interval. Pacing Clin. Electrophysiol..

[9] Glikson M., Burri H., Abdin A., Cano O., Curila K., De Pooter J., Diaz J.C., Drossart I., Huang W., Israel C.W. (2025). European Society of Cardiology clinical consensus statement on indications for conduction system pacing, with special contribution of the European Heart Rhythm Association of the ESC and endorsed by the Asia Pacific Heart Rhythm Society, the Canadian Heart Rhythm Society, the Heart Rhythm Society, and the Latin American Heart Rhythm Society. Europace.

[10] Chung M.K., Patton K.K., Lau C.-P., Forno A.R.D., Al-Khatib S.M., Arora V., Birgersdotter-Green U.M., Cha Y.-M., Chung E.H., Cronin E.M. (2023). 2023 HRS/APHRS/LAHRS guideline on cardiac physiologic pacing for the avoidance and mitigation of heart failure. Heart Rhythm.

[11] Durrer D., van Dam R.T., Freud G.E., Janse M.J., Meijler F.L., Arzbaecher R.C. (1970). Total excitation of the isolated human heart. Circulation.

[12] A Vassallo J., Cassidy D.M., E Marchlinski F., E Buxton A., Waxman H.L., Doherty J.U., E Josephson M. (1984). Endocardial activation of left bundle branch block. Circulation.

[13] Prinzen F.W., Vernooy K., De Boeck B.W.L., Delhaas T. (2011). Mechano-energetics of the asynchronous and resynchronized heart. Heart Fail. Rev..

[14] Huang W., Chen X., Su L., Wu S., Xia X., Vijayaraman P. (2019). A beginner’s guide to permanent left bundle branch pacing. Heart Rhythm.

[15] Chen X., Wu S., Su L., Su Y., Huang W. (2019). The characteristics of the electrocardiogram and the intracardiac electrogram in left bundle branch pacing. J. Cardiovasc. Electrophysiol..

[16] Jastrzębski M., Kiełbasa G., Curila K., Moskal P., Bednarek A., Rajzer M., Vijayaraman P. (2021). Physiology-based electrocardiographic criteria for left bundle branch capture. Heart Rhythm.

[17] Wu S., Chen X., Wang S., Xu L., Xiao F., Huang Z., Zheng R., Jiang L., Vijayaraman P., Sharma P.S. (2021). Evaluation of the Criteria to Distinguish Left Bundle Branch Pacing From Left Ventricular Septal Pacing. JACC Clin. Electrophysiol..

[18] Jastrzębski M., Burri H., Kiełbasa G., Curila K., Moskal P., Bednarek A., Rajzer M., Vijayaraman P. (2022). The V6-V1 interpeak interval: A novel criterion for the diagnosis of left bundle branch capture. Europace.

[19] Perez-Riera A.R., de Abreu L.C., Barbosa-Barros R., Nikus K.C., Baranchuk A. (2016). R-Peak Time: An Electrocardiographic Parameter with Multiple Clinical Applications. Ann. Noninvasive Electrocardiol..

[20] Wu S., Su L., Wang S., Vijayaraman P., Ellenbogen K.A., Huang W. (2019). Peri-left bundle branch pacing in a patient with right ventricular pacing-induced cardiomyopathy and atrioventricular infra-Hisian block. Europace.

[21] Žižek D., Žlahtič T., Mrak M., Ivanovski M., Štublar J., Džananović D.Z., Peterlin J., Cvijić M., Mežnar A.Z. (2025). Conduction system pacing versus biventricular pacing for cardiac resynchronization: The CSP-SYNC randomized single-centre study. Europace.

[22] Wang Y., Zhu H., Hou X., Wang Z., Zou F., Qian Z., Wei Y., Wang X., Zhang L., Li X. (2022). Randomized trial of left bundle branch vs biventricular pacing for cardiac resynchronization therapy. J. Am. Coll. Cardiol..

[23] Vijayaraman P., Sharma P.S., Cano Ó., Ponnusamy S.S., Herweg B., Zanon F., Jastrzebski M., Zou J., Chelu M.G., Vernooy K. (2023). Comparison of left bundle branch area pacing and biventricular pacing in candidates for resynchronization therapy. J. Am. Coll. Cardiol..

[24] Wu S., Su L., Vijayaraman P., Zheng R., Cai M., Xu L., Shi R., Huang Z., Whinnett Z.I., Huang W. (2021). Left bundle branch pacing for cardiac resynchronization therapy: Nonrandomized on-treatment comparison with His bundle pacing and biventricular pacing. Can. J. Cardiol..

[25] Zhu H., Qin C., Du A., Wang Q., He C., Zou F., Li X., Tao J., Wang C., Liu Z. (2024). Comparisons of long-term clinical outcomes with left bundle branch pacing, left ventricular septal pacing, and biventricular pacing for cardiac resynchronization therapy. Heart Rhythm.

[26] Diaz J.C., Sauer W.H., Duque M., Koplan B.A., Braunstein E.D., Marín J.E., Aristizabal J., Niño C.D., Bastidas O., Martinez J.M. (2023). Left bundle branch area pacing versus biventricular pacing as initial strategy for cardiac resynchronization. Clin. Electrophysiol..

[27] Li X., Qiu C., Xie R., Ma W., Wang Z., Li H., Wang H., Hua W., Zhang S., Yao Y. (2020). Left bundle branch area pacing delivery of cardiac resynchronization therapy and comparison with biventricular pacing. ESC Heart Fail..

[28] Pujol-López M., Graterol F.R., Borràs R., Garcia-Ribas C., Guichard J.B., Regany-Closa M., Jiménez-Arjona R., Niebla M., Poza M., Carro E. (2025). Clinical response to resynchronization therapy: Conduction system pacing vs biventricular pacing: The CONSYST-CRT trial. Clin. Electrophysiol..

[29] Guo J., Li L., Xiao G., Ye T., Huang X., Meng F., Li Q., Chen S., Cai B. (2020). Remarkable response to cardiac resynchronization therapy via left bundle branch pacing in patients with true left bundle branch block. Clin. Cardiol..

[30] Tedrow U.B., Miranda-Arboleda A.F., Sauer W.H., Duque M., Koplan B.A., Marín J.E., Aristizabal J.M., Niño C.D., Bastidas O., Martinez J.M. (2024). Sex differences in left bundle branch area pacing versus biventricular pacing for cardiac resynchronization therapy. Clin. Electrophysiol..

[31] Cano Ó., Pérez-Roselló V., Di Marco A., Ramos-Maqueda J., Moriña P., Brouzet T., Rodríguez-Muñoz D., Castro V., Giacoman S., Peñafiel P. (2026). Left bundle branch area vs biventricular pacing for cardiac resynchronization therapy: The LEFT-BUNDLE-CRT trial. Eur. Heart J..

[32] Jastrzębski M., Kiełbasa G., Cano O., Curila K., Heckman L., De Pooter J., Chovanec M., Rademakers L., Huybrechts W., Grieco D. (2022). Left bundle branch area pacing outcomes: The multicentre European MELOS study. Eur. Heart J..

[33] Jastrzębski M., Foley P., Chandrasekaran B., Whinnett Z., Vijayaraman P., Upadhyay G.A., Schaller R.D., Gardas R., Richardson T., Kudlik D. (2024). Multicenter hemodynamic assessment of the LOT-CRT strategy: When does combining left bundle branch pacing and coronary venous pacing enhance resynchronization? Primary results of the CSPOT study. Circ. Arrhythmia Electrophysiol..

[34] Sanchez-Nadales A., Sarkar A., Sleiman J., Sanchez-Nadales A., Alonso M., Bibawy J., Helguera M., Pinski S., Baez-Escudero J. (2025). Safety and performance of the Medtronic 3830 lead in His-bundle and left bundle branch area pacing: A single-center experience. J. Interv. Card. Electrophysiol..

[35] Vijayaraman P., West M., Dresing T., Oren J., Abbey S., Zimmerman P., Bauer R., Butler K., Mangrolia H. (2025). Safety and performance of conduction system pacing: Real-world experience from a product surveillance registry. Heart Rhythm.

[36] Jastrzębski M., Moskal P., Huybrechts W., Curila K., Sreekumar P., Rademakers L.M., Ponnusamy S.S., Herweg B., Sharma P.S., Bednarek A. (2022). Left bundle branch-optimized cardiac resynchronization therapy (LOT-CRT): Results from an international LBBAP collaborative study group. Heart Rhythm.

